# Pilot Study on the Profiling and Functional Analysis of mRNA, miRNA, and lncRNA in the Skeletal Muscle of Mongolian Horses, Xilingol Horses, and Grassland-Thoroughbreds

**DOI:** 10.3390/ani15081123

**Published:** 2025-04-13

**Authors:** Wenqi Ding, Wendian Gong, Tugeqin Bou, Lin Shi, Yanan Lin, Huize Wu, Manglai Dugarjaviin, Dongyi Bai

**Affiliations:** 1 Key Laboratory of Equus Germplasm Innovation (Co-Construction by Ministry and Province), Ministry of Agriculture and Rural Affairs, Hohhot 010018, China; dingwenqi0331@gmail.com (W.D.); gongwendian1996@outlook.com (W.G.); tvgqin@gmail.com (T.B.); 19832607527@163.com (L.S.); linyanan@emails.imau.edu.cn (Y.L.); whz020419@163.com (H.W.); dmanglai@163.com (M.D.); 2 Inner Mongolia Key Laboratory of Equine Science Research and Technology Innovation, Inner Mongolia Agricultural University, Hohhot 010018, China; 3 Equus Research Center, College of Animal Science, Inner Mongolia Agricultural University, Hohhot 010018, China

**Keywords:** Grassland-Thoroughbreds, RNA-seq, skeletal muscle

## Abstract

Muscle fibers, as the fundamental units of muscle tissue, play a crucial role in determining skeletal muscle function through their growth, development, and composition. To investigate changes in muscle fiber types and their regulatory mechanisms in Mongolian horses (MG), Xilingol horses (XL), and Grassland-Thoroughbreds (CY), we conducted histological and bioinformatic analyses on the gluteus medius muscle of these three horse breeds. Immunofluorescence analysis revealed that Grassland-Thoroughbreds had the highest proportion of fast-twitch muscle fibers at 78.63%, while Mongolian horses had the lowest proportion at 57.54%. Whole-transcriptome analysis identified 105 differentially expressed genes (DEGs) in the CY vs. MG comparison and 104 DEGs in the CY vs. XL comparison. Time-series expression profiling grouped the DEGs into eight gene sets, with three sets showing significantly up-regulated or down-regulated expression patterns (*p* < 0.05). Additionally, 280 differentially expressed long non-coding RNAs (DELs) were identified in CY vs. MG, and 213 DELs were identified in CY vs. XL. A total of 32 differentially expressed microRNAs (DEMIRs) were identified in CY vs. MG, while 44 DEMIRs were found in CY vs. XL. Functional enrichment analysis indicated that the DEGs were significantly enriched in essential biological processes, such as actin filament organization, muscle contraction, and protein phosphorylation. KEGG pathway analysis showed their involvement in key signaling pathways, including the mTOR signaling pathway, FoxO signaling pathway, and HIF-1 signaling pathway. Furthermore, functional variation-based analyses revealed associations between non-coding RNAs and mRNAs, with some non-coding RNAs targeting genes potentially related to muscle function regulation. These findings provide valuable insights into the molecular basis for the environmental adaptability, athletic performance, and muscle characteristics in horses, offering new perspectives for the breeding of Grassland-Thoroughbreds.

## Simple Summary

Muscle function is regulated by numerous interacting genes and signaling pathways. To explore the changes in the skeletal muscle types of Mongolian horses (MG), Xilingol horses (XL), and Grassland-Thoroughbreds (CY), immunofluorescence was used to determine that Grassland-Thoroughbreds have the highest proportion of fast-twitch muscle fibers. RNA-seq analysis revealed 105 and 104 differentially expressed genes in the CY vs. MG and CY vs. XL groups, respectively, which were functionally enriched in muscle contraction, the mTOR signaling pathway, and the HIF-1 signaling pathway. Additionally, differentially expressed non-coding RNAs may regulate target genes involved in the establishment of skeletal muscle-specific adaptations. This study contributes to the genomic breeding improvement of the Grassland-Thoroughbred population and provides an important research foundation for understanding its environmental adaptation mechanisms and athletic performance.

## 1. Introduction

Horses hold a unique position in modern society, evolving from being mere production tools to becoming an indispensable part of human culture. Today, horses are primarily used in sporting and recreational activities, with horse racing emerging as one of the most popular competitive events worldwide [1]. Horse racing and breeding industries are widespread globally, spanning at least 71 countries and regions, with more than 500,000 participating horses and a total global prize pool exceeding EUR 3.3 billion [2]. In recent years, with the rapid development of China’s modern equine industry, significant progress has been made in addressing the gap between high-quality racehorses and sport horses. The cultivation of racehorse breeds with distinct Chinese characteristics has become a key objective in the industry’s current development.

The Mongolian horse is one of the oldest surviving horse breeds in the world and is renowned for its exceptional endurance, ability to thrive on coarse forage, and strong disease resistance [3]. As a crucial indigenous equine genetic resource in China, the Mongolian horse is often used as a high-quality parental breed for horse improvement programs. The Xilingol horse, in particular, was developed by using the Mongolian horse as the maternal breeder over 35 years of selective breeding, culminating in its official recognition as a new breed in 1987 [4]. Thoroughbreds are globally recognized for their outstanding athletic performance and racing ability, particularly excelling in specific types of speed competitions. They have long been regarded as the premier racehorse breed [5,6]. To further enhance equine athletic performance, conformation, and speed, Thoroughbreds were introduced in 1995 for crossbreeding with Xilingol horses. After nearly 30 years of continuous selection, this breeding program has produced high-performance offspring, temporarily designated as the Grassland-Thoroughbreds. Due to the distinctive sunburst branding on their hindquarters, they are also known as “Sunflower Horses”. As genetic improvement efforts have progressed, research focus has gradually shifted from merely improving body conformation and speed to investigating changes in muscle fiber types. This characteristic is increasingly being recognized as a key factor influencing both the environmental adaptability and athletic performance of horses.

In mammals, skeletal muscles are composed of different types of muscle fibers, which play a crucial role in motor function and athletic performance [7]. Based on their morphological characteristics and physiological functions, muscle fibers are typically divided into two main types: fast-twitch fibers and slow-twitch fibers [8]. Fast-twitch fibers are rich in glycogen and ATPase, exhibiting strong glycolytic capacity, while slow-twitch fibers contain abundant mitochondria, capillaries, and myoglobin and primarily rely on oxidative metabolism for energy production [9]. The differences between fast- and slow-twitch fibers significantly impact an animal′s performance, influencing breeding strategies for specific traits such as endurance and speed. In recent years, the application of molecular biology techniques to genetic improvement has gained widespread attention. The rapid development of molecular biology and bioinformatics has not only promoted the extensive use of molecular breeding technologies but also advanced research into the regulatory mechanisms of non-coding genes.

Non-coding RNAs play a crucial role in the growth and development of skeletal muscle by regulating gene transcription and translation. Among them, microRNAs (miRNAs) are a class of non-coding RNAs, approximately 22 nucleotides in length, that regulate gene expression at the post-transcriptional level by either inhibiting messenger RNA (mRNA) translation or promoting its degradation [10]. *miRNA-1* and *miRNA-206* are involved in skeletal muscle development and differentiation, promoting the repair of exercise-induced muscle injury [11]. Studies have shown that *miRNA-1* and *miRNA-133* are significantly associated with aerobic exercise and positively correlated with VO_2_ max. They are also associated with hemoglobin levels in the blood and cardiopulmonary health [12,13,14]. Long non-coding RNAs (lncRNAs) are RNA transcripts longer than 200 nucleotides [15]. lncRNAs can influence gene expression through various mechanisms: they may overlap with gene promoter regions to regulate transcription or indirectly modulate gene expression by affecting mRNA stability [16,17]. Additionally, lncRNAs can act as “sponges” for miRNAs, competing for miRNA binding sites on target mRNAs, thereby reducing the regulatory effect of miRNAs on their target mRNAs [18,19]. miRNAs and lncRNAs work synergistically during skeletal muscle contraction and development, playing a critical role in regulating muscle function [20,21]. For example, Ricard identified a new candidate equine long non-coding RNA (*KCNQ1OT1*) through a genome-wide screening for endurance exercise ability, which may be related to cardiac rhythm regulation [22]. Tugeqin Bou identified *FOXO1* upstream of *MSTRG.17944.1* at a distance of 10 kb, suggesting that *FOXO1* may play an important role in muscle energy homeostasis during fasting in horses [23]. The intricate regulatory network of these non-coding RNAs not only reveals the molecular basis of skeletal muscle development but also provides potential molecular targets for the genetic improvement of equine athletic performance in the future. Combining gene editing and multi-omics analysis can significantly improve breeding efficiency.

This study analyzed the expression profiles of mRNA, lncRNA, and miRNA in the skeletal muscle of Mongolian horses, Xilingol horses, and Grassland-Thoroughbreds. It also provides a scientific foundation for the genetic improvement of native Chinese horse breeds, advancing research into horse breeding and athletic traits.

## 2. Materials and Methods

### 2.1. Sample Collection

In this study, skeletal muscle samples were collected from three horse breeds: Mongolian horses (MG), Xilingol horses (XL), and Grassland-Thoroughbreds (CY), which were sourced from the Xilingol Grassland in Inner Mongolia and the Inner Mongolia Grassland-Thoroughbreds Breeding Company. The Grassland-Thoroughbreds were developed by crossbreeding the Xilingol horse with Thoroughbreds. This breeding pathway is illustrated in Supplementary Figure S1. All horses were free-ranging on the grassland. Following standardized procedures, samples were collected by a professional veterinarian under sterile conditions after intravenous anesthesia. Muscle tissue was collected from a depth of approximately 3 cm in the gluteus medius of each horse. The collected samples were immediately washed with phosphate-buffered saline (PBS). Some samples were fixed in formaldehyde solution for subsequent histological analysis, while the remaining samples were rapidly frozen in liquid nitrogen and stored at −80 °C for molecular biological studies.

### 2.2. Library Construction

Total RNA was extracted from the gluteus medius samples of MG, XL and CY using the Trizol reagent (Invitrogen, Carlsbad, CA, USA). RNA purity and integrity were assessed using the Agilent Bioanalyzer 2100 system (Agilent Technologies, Santa Clara, CA, USA), and strand-specific library preparation was employed. RNA sequencing (RNA-seq) libraries were constructed using the NEBNext^®^ Ultra™ Directional RNA Library Preparation Kit (New England Biolabs, Ipswich, MA, USA), while small-RNA sequencing (sRNA-seq) libraries were prepared using the NEBNext^®^ Multiplex Small RNA Library Preparation Kit (New England Biolabs, Ipswich, MA, USA). Library products were then purified using the AMPure XP system (Beckman Coulter, Beverly, MA, USA), and the library quality was evaluated using the Agilent Bioanalyzer 2100 system (Agilent Technologies, Santa Clara, CA, USA). Finally, RNA-seq libraries were sequenced on the Illumina HiSeq 2500 platform, generating 150 bp paired-end sequencing reads. Small-RNA samples were sequenced on the Illumina HiSeq 2500 platform (Illumina, San Diego, CA, USA), generating 50 bp single-end reads.

### 2.3. Mapping and Initial Assembly of RNA-Seq Reads

Raw data from the sequencer were subjected to quality control using Fastp (version 0.20.0) [24]. Quality control included the removal of adapter sequences, exclusion of reads containing ≥ 10% unidentified nucleotides (N), and filtering of low-quality reads with a quality score ≤ Q20. Clean data were then aligned to the horse reference genome (https://www.ncbi.nlm.nih.gov/datasets/genome/GCF_002863925.1/, accessed on 27 November 2020) using Hisat2 (version 2.0.4) [25]. The alignment results were converted into the desired format using Samtools (version 1.19.2), and transcript reconstruction was performed using StringTie (version 2.0) [26], which generated transcript annotations and expression information. Gene expression data were then extracted from the RNA-seq data using the R package Ballgown (version 2.16.0) [27], and the differential expression analysis of gene expression levels was conducted using DESeq2 (version 1.44.0) [28]. The filtering criteria for differentially expressed genes (DEGs) were set as |log2(Fold Change)| > 1 and an adjusted false-discovery rate (FDR) < 0.05 to ensure the statistical reliability of the results.

To identify lncRNAs in different horse breeds, this study utilized tools such as CPAT [29], CNCI [30], and CPC2 [31] for lncRNA prediction. The overlapping results were analyzed for the differential expression analysis of lncRNAs (DELs) using DESeq2 (version 1.24.0). Potential cis-target mRNAs were identified based on the regulatory influence of lncRNAs within the upstream 10 kb and downstream 10 kb regions of the genome, considering protein-coding genes that may be affected by lncRNAs within these regions. The prediction of trans-target mRNAs was based on a Pearson correlation coefficient of r ≥ 0.95 between lncRNAs and mRNAs, which indicated a strong co-expression relationship.

Small-RNA raw data were processed using Cutadapt software (version 3.2) [32], trimming adapter sequences and removing low-quality reads to obtain clean data. The processed data were then assessed for quality using FastQC (version 0.11.5) [33] to ensure that they met the requirements for subsequent analyses. The filtered sequences were aligned to several databases using Bowtie2 (version 1.1.0) [34], including GtRNAdb (http://gtrnadb.ucsc.edu/, accessed on 16 January 2021), Rfam (https://rfam.org/, accessed on 16 January 2021), RepBase (https://www.girinst.org/repbase/, accessed on 16 January 2021), and SILVA (https://www.arb-silva.de/, accessed on 16 January 2021). This process helps remove non-target small RNA types and repetitive sequences. The filtered data were then aligned to the reference genome using mapper.pl in miRDeep2 (version 0.0.8) [35], and miRDeep2 was used to identify known miRNAs. Finally, differential expression analysis was performed using DESeq2 to identify significantly differentially expressed miRNAs (DEMIRs) between the horse breeds. To further predict miRNA target genes, MiRanda (https://www.miranda.software/) [36] and TargetScan (targetscan.org) [37] were used to predict conserved target genes matching each miRNA seed region.

### 2.4. Functional Enrichment Analysis

To evaluate the biological functions and potential mechanisms of DEGs and non-coding RNA target genes, Gene Ontology (GO) and Kyoto Encyclopedia of Genes and Genomes (KEGG) enrichment analyses were performed using the DAVID online tool (https://david.ncifcrf.gov/, accessed on 30 November 2024). A significance threshold of FDR < 0.05 was applied to identify significantly enriched functional categories and signaling pathways, elucidating the biological roles of the DEGs and target genes.

### 2.5. Co-Expression Network Construction

To identify hub genes that play key roles in muscle function, this study conducted a protein–protein interaction (PPI) network analysis of DEGs using the STRING protein interaction database (https://string-db.org/, accessed on 21 December 2024). The generated network file was imported into Cytoscape (version 3.7.0) for visualization, editing, and in-depth network analysis. Additionally, to explore the functions of key lncRNAs, we analyzed the correlation between trans-target mRNAs and lncRNAs. Special attention was given to the potential regulatory relationships between DELs and DEGs involved in muscle fiber structure formation and contraction processes. An lncRNA-mRNA co-expression network was constructed to reveal their critical roles in regulating muscle function. Using the same method, an miRNA-mRNA co-expression network was constructed to identify the regulatory roles of specific DEMIRs.

### 2.6. Expression Trend Analysis

Trend analysis is an important method in gene expression studies. In this study, gene expression levels were preprocessed using log2 normalization in STEM (version 1.3.13) [38]. The software was configured to generate the eight most representative expression patterns by default, with a minimum fold-change threshold of 2. Clustering patterns with a *p*-value ≤ 0.05 were considered statistically significant.

### 2.7. Quantitative Real-Time PCR

To validate the accuracy of the sequencing results in muscle samples from different horse breeds, quantitative real-time PCR (qRT-PCR) was performed for both mRNA and lncRNA. The qRT-PCR primers were designed by Shanghai Sangon Biotech (Shanghai, China) (Supplementary Table S1). Total RNA was reverse transcribed into cDNA using the HiScript^®^ II qRT SuperMix for qPCR kit (Vazyme, Nanjing, China), following the manufacturer’s instructions. GAPDH (glyceraldehyde-3-phosphate dehydrogenase) was used as the endogenous reference gene [39]. qRT-PCR was conducted using the CFX96 Real-Time PCR Detection System (Bio-Rad, Hercules, CA, USA) with SYBR^®^ Premix Ex Taq™ II (TaKaRa, Osaka, Japan). Each sample was tested in triplicate to ensure data reliability and accuracy.

Due to the short sequence length of miRNAs, a stem–loop miRNA first-strand cDNA synthesis kit (Vazyme, Nanjing, China) was used to synthesize cDNA from total RNA, followed by real-time qPCR on the CFX96 Real-Time PCR Detection System. hsa-miR-26a was used as the endogenous control gene [40,41]. Detection was performed using specific primers and the miRNA Universal SYBR qPCR Master Mix. The qPCR results were analyzed using the 2^(−ΔΔCt)^ method to calculate Ct value changes across muscle samples from the different horse breeds, with an FDR < 0.05 considered statistically significant.

## 3. Results

### 3.1. Muscle Fiber Types in Three Horse Breeds

**Figure 1:**
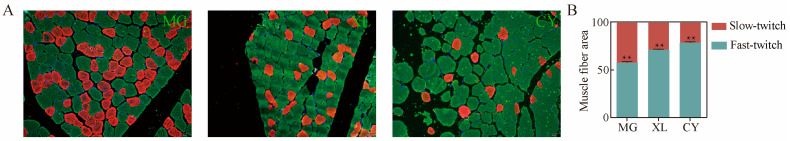
Proportion of muscle fiber types in Mongolian horses, Xilingol horses, and Grasslands-Thoroughbreds. (**A**) Immunofluorescence staining. Bar = 50 μm. The myonuclei in the gluteus medius are stained blue, fast muscle fibers are green, and slow muscle fibers are red. (**B**) Proportion of muscle fiber types; ** indicates a highly significant difference with *p* < 0.01.

Immunofluorescence analysis revealed distinct differences in the fluorescence signals between fast-twitch and slow-twitch muscle fibers. Fast-twitch fibers exhibited green fluorescence, while slow-twitch fibers displayed red fluorescence (Figure 1A). Analysis showed that the proportion of fast-twitch fibers in the gluteus medius muscle was 57.54% in MG, 71.09% in XL, and 78.63% in CY. The differences among the groups were statistically significant (*p* < 0.05) (Figure 1B).

### 3.2. Differential Expression Analysis of mRNA

**Figure 2:**
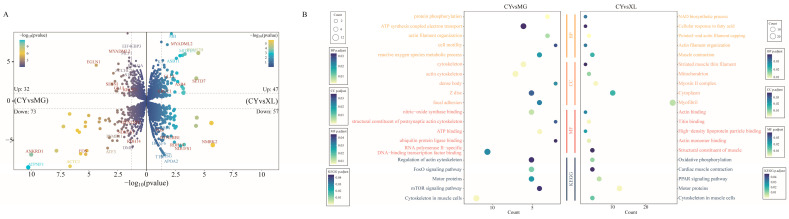
Expression and pathway analysis of differentially expressed mRNAs (DEGs) in MG, XL, and CY. (**A**) Volcano plots of DEGs for CY vs. MG (**left panel**) and CY vs. XL (**right panel**). (**B**) GO and KEGG functional enrichment results of DEGs for CY vs. MG (**left panel**) and CY vs. XL (**right panel**). The circle size indicates the number of differentially expressed genes annotated, and the circle color represents the *p*-value.

To investigate differences in the gene expression patterns in the gluteus medius muscle across different horse breeds, we used CY as the control group. The sequencing generated raw reads ranging from 79.47 million to 96.90 million. After quality filtering, the Q30 values ranged from 94.13% to 95.29%, resulting in clean reads between 79.13 million and 96.50 million. Of these, 96.24% to 97.27% of the reads were successfully mapped to the reference genome (Supplementary Table S2). To explore the regulatory roles of the mRNAs in the different horse breeds, we analyzed the DEGs among MG, XL, and CY. A total of 105 DEGs were identified in CY vs. MG, with 73 down-regulated genes and 32 up-regulated genes. For CY vs. XL, 104 DEGs were identified, including 57 up-regulated and 47 down-regulated genes (Figure 2A) (Supplementary Table S3). Nine genes were commonly up-regulated in both comparisons: *SETD7*, *ASB4*, *KRCC1*, *SESN1*, *EGLN1*, *MYADML2*, *GLUL*, *LOC102149379*, and *LOC100054521*. To further elucidate the functions of these DEGs, we performed GO and KEGG enrichment analyses for functional annotation and classification.

In the CY vs. MG groups, GO enrichment analysis of the DEGs revealed significant involvement in metabolic processes, signal transduction, tissue development, and the stress response (Figure 2B). Significantly enriched GO terms included the regulation of transepithelial transport (GO:0150111), cardiac muscle tissue morphogenesis (GO:0055008), and the regulation of synaptic vesicle endocytosis (GO:1900242), among others. KEGG pathway analysis identified a total of 27 enriched signaling pathways that were primarily associated with disease, signal transduction, muscle contraction, and energy metabolism. Notably, only eight up-regulated DEGs were enriched, with several pathways related to muscle fiber development, contraction, and energy metabolism identified. These included the mTOR signaling pathway (ecb04150), the FoxO signaling pathway (ecb04068), regulation of the actin cytoskeleton (ecb04810), the HIF-1 signaling pathway (ecb04066), and motor proteins (ecb04814).

In the CY vs. XL groups, approximately 57% of the DEGs were enriched in GO terms primarily associated with biological processes related to muscle contraction and energy metabolism (Figure 2B). The enriched GO terms included muscle contraction (GO:0006936), actin filament organization (GO:0007015), and protein stabilization (GO:0050821). KEGG pathway enrichment analysis revealed that the DEGs were mainly involved in pathways related to cytoskeletal structure, metabolic balance, cellular physiological functions, and disease defense. Notable pathways included ferroptosis (ecb04216), the cytoskeleton in muscle cells (ecb04820), and cardiac muscle contraction (ecb04260) (Supplementary Table S6).

### 3.3. The Role of lncRNA Changes

**Figure 3:**
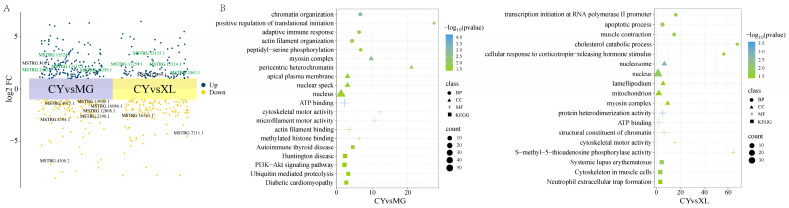
Expression and pathway analysis of differentially expressed lncRNAs (DELs) in MG, XL, and CY. (**A**) Scatter plots of DELs for CY vs. MG and CY vs. XL. (**B**) GO and KEGG functional enrichment results of target genes within 10 kb upstream or downstream of DELs for CY vs. MG and CY vs. XL. The circle size indicates the number of differentially expressed genes annotated.

In the CY vs. MG groups, a total of 280 DELs were identified, including 121 down-regulated and 159 up-regulated DELs (Figure 3A). By analyzing the potential cis-target genes of these DELs within a 10 kb upstream and downstream range, GO enrichment revealed that these genes were primarily involved in biological processes related to cell growth and death, the immune response, transcriptional regulation, cytoskeletal organization, and movement (Figure 3B). Notably, key enriched GO terms included actin filament organization (GO:0007015), protein phosphorylation (GO:0006468), and actin-mediated cell contraction (GO:0070252). These pathways play crucial roles in coordinating muscle contraction, regulating myogenesis, and influencing the locomotor capacity. KEGG pathway analysis identified five significantly enriched signaling pathways, most of which were related to the immune response and disease. Among them, two pathways were specifically associated with muscle mass and contraction: the PI3K-Akt signaling pathway (ecb04151) and ubiquitin-mediated proteolysis (ecb04120). These pathways collectively contribute to maintaining muscle health, function, and adaptive responses.

In the CY vs. XL groups, 213 DELs were identified, including 125 down-regulated and 88 up-regulated DELs (Supplementary Table S4). GO and KEGG enrichment analyses indicated that the target genes of these DELs were involved in pathways related to stress response, metabolism, and immunity. Notably, two pathways were closely associated with muscle function: muscle contraction (GO:0006936) and the cytoskeleton in muscle cells (ecb04820) (Supplementary Table S7).

### 3.4. The Role of MicroRNA Changes

**Figure 4:**
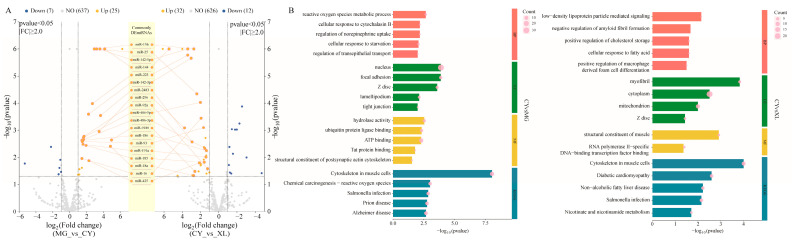
Expression and pathway analysis of differentially expressed miRNAs (DEMIRs) in MG, XL, and CY. (**A**) Scatter plots of DEMIRs for CY vs. MG and CY vs. XL. The connecting lines represent the up-regulated DEMIRs shared between the two groups. (**B**) GO and KEGG functional enrichment results of DEMIR target genes for CY vs. MG and CY vs. XL. The circle size indicates the number of differentially expressed genes annotated.

To investigate the regulatory role of miRNAs in skeletal muscle across the different horse breeds, differential expression analysis was performed. In the CY vs. MG groups, 32 DEMIRs were identified, including 7 down-regulated and 25 up-regulated miRNAs (Figure 4A). By analyzing the negative correlation between the miRNA and mRNA expression levels, we predicted the target genes of these miRNAs and conducted functional annotation and enrichment analysis. The GO enrichment results indicated that the target genes were primarily associated with energy metabolism, signal transduction, cell structure, and development (Figure 4B). Notably, the glycogen biosynthetic process (GO:0005978) was significantly enriched. Glycogen serves as an efficient energy storage molecule, allowing for the rapid release of energy to support intense muscle contractions and movement. KEGG pathway analysis further revealed that these target genes were significantly enriched in 27 signaling pathways that are involved in key biological processes related to metabolism, disease, cell communication, and endocrine regulation.

In the CY vs. XL groups, 44 DEMIRs were identified, with 32 up-regulated and 12 down-regulated miRNAs (Supplementary Table S5). The biological pathways primarily involved lipid metabolism, cholesterol storage, fatty acid responses, and NAD synthesis, all of which contribute to the cellular energy balance, immune response, and other physiological processes. KEGG analysis revealed significant enrichment of signaling pathways related to immune diseases and energy metabolism (Supplementary Table S8). These pathways not only influence muscle structure, contraction, and stretching but are also closely linked to muscle repair, regeneration, and response to mechanical stress.

### 3.5. Generation of the lncRNA-mRNA Co-Expression Network

**Figure 5:**
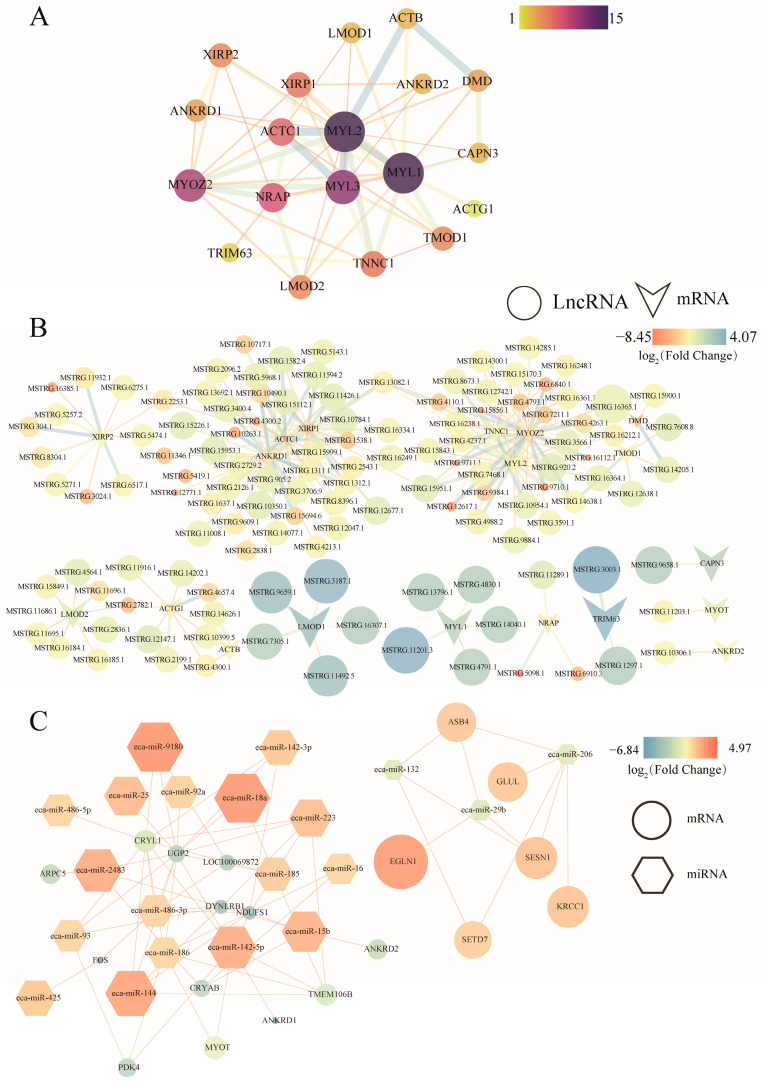
Co-expression networks of the CY vs. MG and CY vs. XL groups that are related to muscle contraction and muscle cytoskeleton. (**A**) Gene network of muscle contraction and structure-related genes in both groups. (**B**) Co-expression network of DLEs related to muscle structure and contraction and their targeted DEGs. The network is based on the Pearson correlation coefficient (r ≥ 0.95) between the DELs and DEGs. Quadrilaterals represent the target DEGs, and circles represent the DELs. (**C**) Common DEMIRs between CY vs. MG and CY vs. XL targeting DEGs, with hexagons representing DEMIRs and circles representing the targeted DEGs.

The PPI analysis revealed key genes involved in muscle cytoskeleton organization and muscle contraction processes, providing insights into the genetic basis for the different athletic ability and muscle function across the different horse breeds. Genes associated with the muscle cell cytoskeleton and contraction, which were enriched in both the CY vs. MG and CY vs. XL groups, were submitted to the STRING database. Network analysis and visualization were performed using the Cytoscape software(version 3.7.0). The PPI network analysis highlighted that the myosin light chain family genes MYL1, MYL2, and MYL3 play crucial roles in muscle fiber cytoskeletal organization and contraction (Figure 5A). Furthermore, these genes are key regulators of muscle cytoskeleton integrity and contraction function.

To identify the key DELs involved in the regulation of muscle system processes, this study integrated coding and non-coding RNAs related to the muscle cell cytoskeleton and contraction from the two groups. A total of 125 DELs were found to target 19 DEGs, which led to the construction of a muscle-function-related lncRNA-mRNA co-expression network with 228 connections (Figure 5B).

To identify specific DEMIR regulatory relationships, this study integrated 19 DEMIRs up-regulated in the CY group and 3 common DEMIRs shared by the MG and XL groups, targeting 20 DEGs. Among these, it was found that miR-132, miR-206, and miR-132 can target genes such as *EGLN1*, *SETD7*, *SESN1*, and *ASB4* (Figure 5C).

### 3.6. Expression Trend Analysis

**Figure 6:**
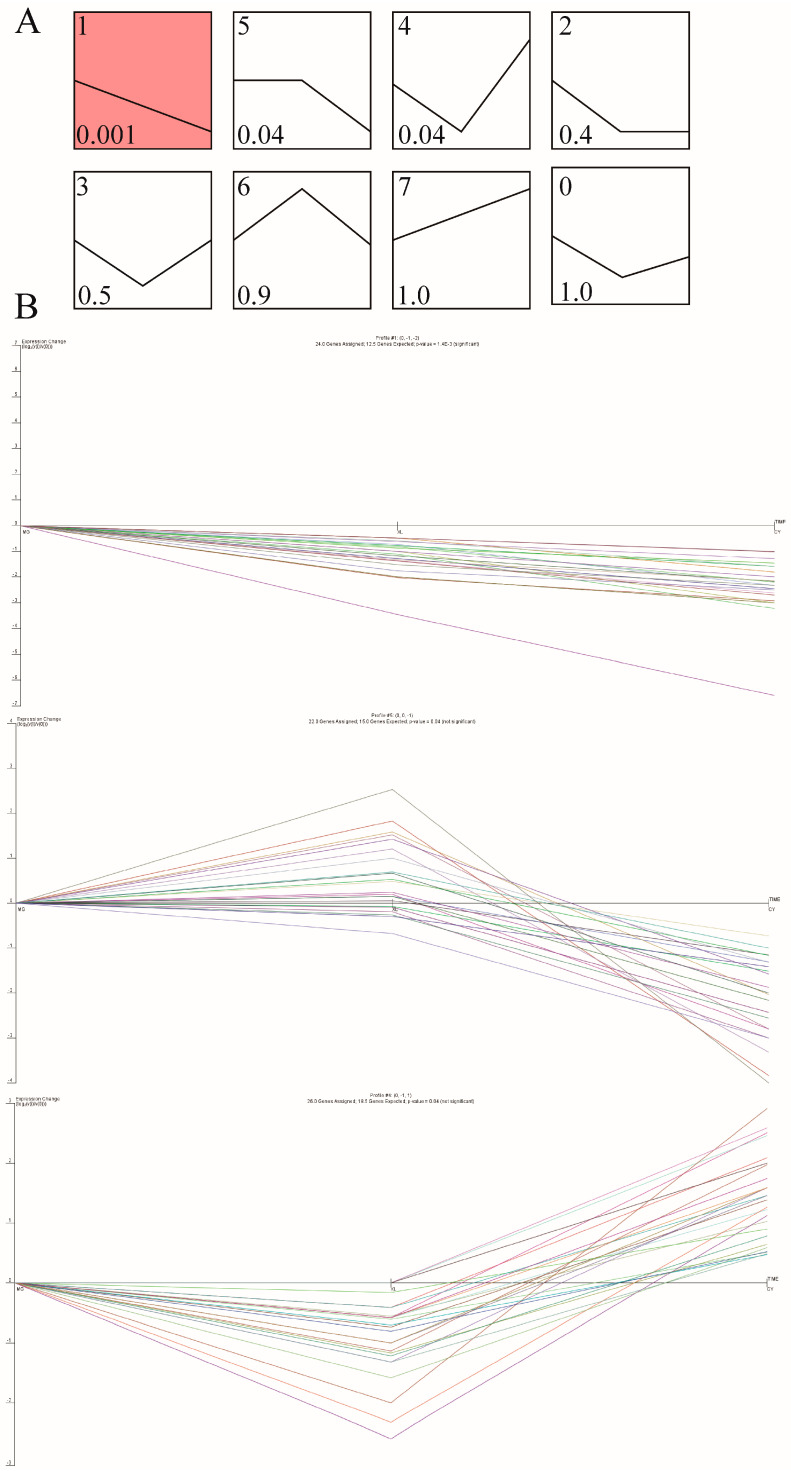
STEM analysis of the DEGs’ expression profiles. (**A**) Trend analysis of the different expression genes; color intensity denotes the level of enrichment. (**B**) Three significant clusters of DEG profiles across all three stages. Each color represents the expression pattern of a single gene.

A total of 184 DEGs from the CY vs. MG and CY vs. XL groups were clustered based on their expression trends across three stages (Figure 6A). Eight distinct expression patterns were identified, of which three were significantly enriched: two down-regulated patterns (Pattern 1 and Pattern 5; *p* < 0.05) and one up-regulated pattern (Pattern 4; *p* < 0.05). Pattern 1, Pattern 5, and Pattern 4 contained 24, 22, and 26 DEGs, respectively. This included 28 up-regulated DEGs and 46 down-regulated DEGs (Figure 6B). These results reveal the dynamic changes in gene expression in skeletal muscle during breed development, providing a reliable dataset for future candidate gene screening in breeding programs.

### 3.7. Validation of DE Coding RNAs and DE Non-Coding RNAs

**Figure 7:**
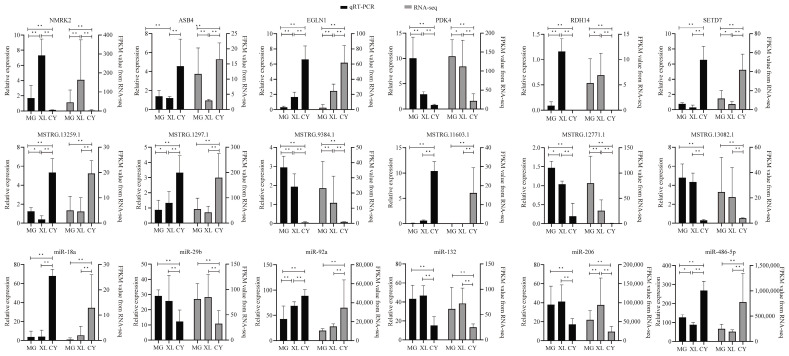
Six differentially expressed genes (DEGs), six differentially expressed lncRNAs, and six differentially expressed miRNAs were validated by qRT-PCR. “*” was considered a significant difference (*p* < 0.05); “**” was considered a highly significant difference (*p* < 0.01). Black represents the qRT-PCR related expression, and gray represents the Fragments Per Kilobase of transcript per Million mapped reads (FPKM) values from the RNA-seq.

To validate the expression of DEGs, DEMIRs, and DELs in the different horse breeds, a total of 18 genes were chosen for validation. These included six mRNAs (*NMRK2*, *PDK4*, *RDH14*, *EGLN1*, *SETD7*, *ASB4*), six lncRNAs (*MSTRG.12771.1*, *MSTRG.13082.1*, *MSTRG.16340.1*, *MSTRG.11603.1*, *MSTRG.1297.1*, *MSTRG.13259.1*), and six miRNAs (*miR-132*, *miR-206*, *miR-29b*, *miR-18a*, *miR-92a*, *miR-486-5p*). The expression levels of these genes were validated using qRT-PCR, and the observed expression patterns were found to be consistent with the results from the RNA-seq and sRNA-seq analyses. Significance was determined based on an FDR < 0.05 (Figure 7).

## 4. Discussion

The most common effect in hybrid breeds is hybrid vigor, where the biological and physiological traits of hybrid organisms surpass those of their parents [42]. To improve horse performance, many breeders are choosing to crossbreed horses with superior traits [43,44]. The athletic ability of horses offers a valuable model for understanding the physiological and molecular mechanisms of exercise adaptation. Skeletal muscle is key for athletic performance, and fast muscle fibers constitute approximately 88% of the hind limb muscle in Thoroughbreds [45], likely contributing to their powerful explosive strength. In this study, the Grassland-Thoroughbreds, a hybrid of the Xilingol horse and the Thoroughbred, contains 78.63% fast muscle fibers and is significantly larger than other horses. This is likely due to the larger diameter of the fast muscle fibers. Compared with the Mongolian and Xilingol horses, this suggests that the Grassland-Thoroughbred combines the explosive power of the Thoroughbred with the adaptive traits from hybridization.

Muscle development is a complex biological process that is regulated by a variety of interacting genes and signaling pathways [46]. In both the CY vs. MG and CY vs. XL comparisons, nine common genes were found to be highly expressed in CY. Gene expression trend analysis revealed that these genes consistently exhibited an up-regulation trend throughout the breeding process. These genes may play a critical role in the specific physiological functions or adaptations in CY. Further analysis revealed that five of these genes were enriched in 11 pathways, with only the *SESN1* and *ASB4* genes being associated with reactive oxygen species metabolism, cellular response to leucine starvation, and protein ubiquitination. *ASB4*, an ankyrin repeat and SOCS-box-containing ubiquitin ligase, promotes angiogenesis and myogenesis in mice by degrading the protein ID2 (DNA binding inhibitor 2) [47]. In chickens, *RPL3L* may regulate muscle growth and development by influencing *ASB4* expression [48]. *SESN1*, the earliest discovered protein in the Sestrin family, is induced by environmental stressors such as oxidative stress, DNA damage, hypoxia, and starvation [49,50,51] and plays a protective role in metabolic disorders [52]. When *SESN1* is overexpressed, its levels increase in both fast and slow muscles, helping to maintain muscle strength and protect muscles from aging-related atrophy [53]. Sestrin is considered a key regulator in exercise metabolism. Acute exercise induces the expression of Sestrin1 [54], whereas prolonged inactivity reduces the expression levels of both Sestrin1 and Sestrin3 in mice [53]. *SESN1* plays a pivotal role in improving insulin sensitivity, enhancing exercise endurance, regulating exercise metabolism, and increasing respiratory efficiency [55]. Additionally, *SESN1* protects skeletal muscles from aging via the longevity gene *FOXO3*, thus slowing the aging process in skeletal muscles [56]. In both groups, *MSTRG.7305.1*, *MSTRG.11492.5*, *MSTRG.9659.1*, *MSTRG.3187.1*, *MSTRG.13699.1*, and *MSTRG.2063.1* were found to be positively correlated with *SESN1* expression. These DELs and their target genes may provide a metabolic advantage to the Grassland-Thoroughbreds during prolonged running and high-intensity exercise. This effect is likely achieved through regulation of the intracellular energy balance and oxidative stress protection, which enhances the respiratory efficiency and supports superior endurance and explosive power. Similarly, two genes, *EGLN1* and *SETD7*, were found to be enriched in two signaling pathways in the KEGG analysis: the HIF-1 signaling pathway and the FoxO signaling pathway. *EGLN1* is a key participant in the oxygen-sensing pathway and serves as the primary prolyl hydroxylase for HIF-α [57,58]. The absence of *EGLN1* results in elevated protein levels of *HIF-1α* and *HIF-2α*, which activate the transcription of target genes, significantly increasing serum levels of Vegf-a and renal Epo mRNA [59,60]. The regulation of *HIF-1α/2α* by *EGLN1* is essential for maintaining blood oxygen concentrations, promoting angiogenesis, and supporting erythropoiesis, all of which are critical physiological processes. Studies have demonstrated that EGLN1 can improve oxygen delivery or the oxygen utilization capacity during exercise in populations residing at high altitudes [61]. Its deficiency leads to a significant increase in skeletal muscle capillary density and induces a shift in the skeletal muscle phenotype toward type I fibers [62]. Its polymorphism is more prevalent among endurance athletes and may be linked to superior aerobic capacity [63]. Moreover, HIF-1 not only plays a key role under hypoxic conditions but also in cold environments. In cold conditions, HIF-1 plays a role in adaptation by increasing its protein levels, DNA-binding activity, and transcriptional response [64,65]. The Thoroughbred typically resides at an altitude of around 300 m, with an average annual temperature of 10 °C [66]. In contrast, the Grassland-Thoroughbred, a hybrid of the Thoroughbred, lives at higher altitudes (above 1000 m), where the average temperature is 0 °C and the average minimum temperature reaches −20 °C [67]. The absence of *EGLN1* plays a crucial role in improving the oxygen supply and utilization capacity in horses by activating the HIF-1 signaling pathway. It promotes the generation of endurance-type muscle fibers, enhancing exercise endurance and recovery capabilities. This enables horses to perform better in long-duration, high-intensity exercise, particularly in high-altitude environments or endurance-based competitions. Another enriched pathway, the FoxO signaling pathway, plays a critical role in regulating skeletal muscle atrophy. It primarily induces the ubiquitin ligase Atrogin-1, which leads to muscle atrophy [68]. *SETD7*, a gene within this pathway, regulates the proliferation and differentiation of muscle stem cells (MuSCs) and is closely associated with muscle fiber development and regenerative capacity [69]. The study found that silencing *SETD7* inhibits the Hippo signaling pathway, increases nuclear expression of HIF-1α, and promotes the glycolytic pathway [70]. *MSTRG.1884.1* is positively correlated with *SETD7* expression in both groups. The high expression level of these genes likely contributes to the superior endurance, muscle recovery, and exercise efficiency of the Grassland-Thoroughbreds. In particular, in cold, high-altitude, and low-oxygen environments, these genes help the horse maintain stable physiological functions, enhancing its athletic performance and adaptability to extreme environmental conditions.

PPI analysis has revealed that MYL family genes play an important role in muscle function, with *MYL1* being a specific and early marker for fast muscle cells in zebrafish [71]. The high expression levels of *MYL1* in the Grassland-Thoroughbreds are consistent with the muscle fiber immunofluorescence results, which show a higher proportion of fast muscle fibers in these horses. Although *MYL1* has more nodes in the PPI network, it is only associated with five lncRNAs in the lncRNA-mRNA co-expression network. This suggests that the expression of *MYL1* in the Grassland-Thoroughbreds is primarily regulated by direct transcription factors or protein interactions. This could indicate a more straightforward regulation of *MYL1*’s role in muscle function, possibly by factors that specifically govern fast-muscle-fiber development and athletic performance.

Skeletal muscle function in mammals is precisely regulated by various miRNAs. In this study, analysis of the CY vs. MG and CY vs. XL groups revealed that approximately three-quarters of the DEMIRs were up-regulated in CY. This suggests that these miRNAs may play a key role in regulating skeletal muscle metabolism, muscle growth, tissue repair, and fatigue resistance in Grassland-Thoroughbreds, helping them better adapt to the environment. Among the up-regulated miRNAs, *miR-486-5p* is closely related to exercise endurance and muscle function [72]. Studies have shown that after intense exercise, the expression level of *miR-486-5p* rapidly decreases [73]. *miR-486-5p* has been identified as a key regulator in the PI3K-AKT signaling pathway. Exercise promotes the release of *miR-486-5p* in exosomes from skeletal muscle, activating protective networks in the cardiovascular system [74]. In the CY vs. XL comparison, *miR-499-5p* was specifically expressed in slow muscle fibers and was significantly up-regulated in Xilingol horses. *miR-499-5p* is a key miRNA regulating myosin and plays a significant role in muscle-fiber-type switching, making it an important biomarker for muscle diseases [75]. Research has shown that knocking out *miR-499-5p* alters the muscle fiber types from slow to fast [76]. In this study, *miR-499-5p* was predicted to target *CAPN3*. Studies have shown that mutations in *CAPN3* are associated with the muscle fiber composition in chickens and pigs [77,78]. Furthermore, overexpression of *miR-499-5p* led to the down-regulation of *CAPN3*, indicating that *miR-499-5p* plays a role in the regulation of muscle fiber type [75]. In addition, *miR-499-3p*, which is derived from the same precursor miRNA as *miR-499-5p*, can regulate the expression of nicotinamide phosphoribosyltransferase (*NAMPT*), thereby promoting the proliferation and differentiation of myoblasts, activating the fast-twitch muscle phenotype, and modulating the AMPK signaling pathway [79]. During skeletal muscle contraction, *NAMPT* regulates mitochondrial biogenesis through NAD metabolism and calcium-binding proteins [80], which may enhance the energy supply and athletic performance by promoting mitochondrial biogenesis and improving metabolic efficiency. In the CY vs. MG comparison, *miR-182* was found to be highly expressed in fast muscle fibers and negatively correlated with blood glucose concentrations. Knockout of *miR-182* leads to muscle atrophy, a shift from fast to slow muscle fibers, and impaired glucose metabolism [81]. Additionally, *miR-182* targets *EGLN1* (also known as *PHD2*) [82], and over expression of *miR-182* enhances angiogenesis by increasing the expression levels of HIF1α and its target gene *VEGF* [83]. By regulating the stability and function of HIF1α, *miR-182* helps horses improve their adaptation to hypoxia. These findings suggest that miRNAs comprehensively regulate skeletal muscle function, metabolism, repair, angiogenesis, and exercise adaptation in Grassland-Thoroughbreds. They play a crucial role in helping the horses cope with hypoxic and cold environments, enhancing their endurance, fatigue resistance, and overall health. These results provide important molecular evidence for genetic breeding, improving athletic performance, and preventing related diseases in Grassland-Thoroughbreds.

ncRNAs typically exert regulatory functions indirectly by modulating target protein-coding genes. lncRNAs play key roles in regulating muscle fiber support, contraction, adaptation, and transformation. In this study, Grassland-Thoroughbreds exhibited fewer up-regulated mRNAs and more up-regulated non-coding RNAs in both groups. A co-expression network of DELs and their target genes was constructed. This network revealed that the XL and MG showed down-regulation, with a total of 125 DELs targeting 19 muscle system process-related mRNAs. These findings suggest that these lncRNAs may positively regulate target mRNAs involved in muscle function in horses. Among the identified lncRNAs, 32 were found to target *ANKRD1* and *MYL2*. *MSTRG.2838.1*, *MSTRG.10263.1*, and *MSTRG.12771.1* were positively correlated with *ANKRD1* expression, which was down-regulated in both the CY vs. MG and CY vs. XL groups. *ANKRD1* is preferentially expressed in type I muscle fibers and transmits signals from the myofibril to the nucleus, especially in response to muscle stress [84,85]. Endurance training significantly increases *ANKRD1* mRNA levels [86]. Similarly, *MSTRG.4237.1*, *MSTRG.15951.1*, and *MSTRG.10954.1* were positively correlated with *MYL2* expression, which was down-regulated only in the XL. *MYL2* is essential for regulating myosin ATPase activity in smooth muscle [87] and plays a vital role in muscle growth and contraction [88]. Additionally, 13 lncRNAs were found to target four genes, with 5 lncRNAs targeting both *LMOD1* and *MYL1*. *LMOD1* is expressed in muscle stem cells and increases in abundance during skeletal muscle regeneration, playing a key role in myotube formation [89]. *MYL1* is also crucial for skeletal muscle development [90]. These findings suggest that the regulation of these genes by lncRNAs may be essential for muscle function and adaptation in Grassland-Thoroughbreds, particularly under extreme conditions. Due to the limitation of the small sample size, the conclusions regarding changes in muscle fiber types and their regulatory mechanisms in this study are preliminary. Although the reliability of the results was enhanced by applying a strict differential expression threshold (FDR < 0.05), the small sample size may affect the detection power of low-abundance transcripts, and the current sample size may underestimate the complexity of some regulatory networks. Future studies should expand the sample size to validate key lncRNA-mRNA interactions and further explore the molecular mechanisms underlying breed differences. This preliminary study not only helps to elucidate the molecular basis of horse environmental adaptability, exercise performance, and muscle characteristics but also provides a new conceptual framework for understanding the regulatory mechanisms of skeletal muscle function during breeding.

## 5. Conclusions

This study examined the proportions of fast and slow muscle fibers across three horse breeds, with the Grassland-Thoroughbreds having the highest proportion of fast muscle fibers. It also analyzed skeletal muscle transcriptomic changes, identifying 184 DEGs. Notably, genes such as *SESN1*, *ASB4*, *EGLN1*, and *SETD7* were found to regulate metabolism, environmental adaptability, and athletic performance. Additionally, the study explored non-coding RNAs, identifying miRNAs as regulators of muscle metabolism, angiogenesis, and exercise adaptation. DELs may contribute to skeletal-muscle-specific adaptations by regulating their target genes. These findings, encompassing both coding and non-coding molecular elements, offer valuable insights for genomic breeding improvements in the Grassland-Thoroughbred population and provide a critical foundation for understanding their environmental adaptation mechanisms and athletic performance.

## Data Availability

Sequence data that support the findings of this study have been deposited in the National Center for Biotechnology Information with the primary accession code PRJNA1218016.

## Supplementary Materials

The following supporting information can be downloaded at https://www.mdpi.com/article/10.3390/ani15081123/s1. Supplementary Table S1: Primers for qRT-PCR; Supplementary Table S2: Quality control of the RNA sequencing results from skeletal muscle; Supplementary Table S3: Differentially expressed mRNA between the different groups; Supplementary Table S4: Differentially expressed lncRNA between the different groups; Supplementary Table S5: Differentially expressed miRNA between the different groups; Supplementary Table S6: Functional enrichment of differentially expressed genes; Supplementary Table S7: Functional enrichment of differentially expressed lncRNA target genes; Supplementary Table S8: Functional enrichment of differentially expressed miRNA target genes; Supplementary Table S9: The correlation coefficient between the qPCR and RNA-seq data; Supplementary Figure S1: Breeding technology roadmap for the Grassland-Thoroughbred.
